# Particulated Costal Hyaline Cartilage Allograft and Microdrilling Combined with High Tibial Osteotomy Improves Early Pain Outcomes in Patients Suffering from Medial Knee Osteoarthritis with Full-Thickness Cartilage Defects: A Randomized Controlled Trial

**DOI:** 10.3390/medicina62020289

**Published:** 2026-02-01

**Authors:** Gi Beom Kim, Oog-Jin Shon, Sang-Woo Jeon

**Affiliations:** 1 Department of Orthopedic Surgery, Yeungnam University College of Medicine, Hyeonchungno 170, Nam-gu, Daegu 42415, Republic of Korea; donggamgb@hanmail.net (G.B.K.); maestro-jin@hanmail.net (O.-J.S.); 2 Department of Orthopedic Surgery, Ewha Womans University Seoul Hospital, Seoul 07804, Republic of Korea

**Keywords:** particulated costal allocartilage, high tibia osteotomy, MegaCarti, microfracture

## Abstract

*Background and Objectives*: While particulated costal hyaline cartilage allograft (PCHCA) combined with microdrilling demonstrates superior cartilage regeneration compared to microdrilling alone in high tibial osteotomy (HTO), the impact on early clinical recovery remains uncertain. The aim of this study is to compare early clinical outcomes (within 6 months) between microdrilling alone versus combined particulated costal hyaline cartilage allograft (PCHCA) with microdrilling in medial open-wedge high tibial osteotomy (MOWHTO) for medial compartment osteoarthritis, and to investigate age-related differences in treatment response. *Materials and Methods*: This prospective, dual-center, randomized controlled trial with blinded outcome assessment enrolled 64 patients (33 treatment and 31 control) undergoing MOWHTO with medial femoral condyle cartilage defects (ICRS III-IV, ≥200 mm^2^). The treatment group received PCHCA implantation combined with microdrilling, while the control group received microdrilling alone. Patients and outcome assessors were blinded to group allocation. Primary outcomes were KOOS-Pain and VAS scores at 12 and 24 weeks. Age-stratified analysis compared patients ≤ 60 years (n = 44) versus > 60 years (n = 20) *Results*: The treatment group showed significantly superior KOOS-Pain scores at 12 weeks (70.6 vs. 61.6, *p* = 0.014) and 24 weeks (82.9 vs. 71.5, *p* = 0.011), with corresponding VAS improvements (*p* = 0.010 and *p* = 0.004). Age-stratified analysis revealed patients ≤ 60 years achieved comparable outcomes regardless of treatment (*p* = 0.574), while patients > 60 years demonstrated significantly superior outcomes with PCHCA (KOOS-Pain improvement: 24.7 vs. 17.9 points, *p* = 0.012). BMI ≥ 26 kg/m^2^ significantly predicted reduced odds of achieving MCID for both pain (OR 0.88, *p* = 0.028) and ADL (OR 0.80, *p* = 0.003). *Conclusions*: PCHCA combined with microdrilling provides superior early pain relief compared to microdrilling alone in MOWHTO, with effects most pronounced in patients > 60 years. Age-stratified treatment selection and BMI optimization should be considered to maximize outcomes.

## 1. Introduction

High tibial osteotomy (HTO) has been widely recognized as an effective treatment for patients with medial compartment osteoarthritis (OA) and varus malalignment by shifting the weight-bearing line laterally [1]. However, despite this lateral shift, residual weight-bearing on the medial compartment persists, and the degree of medial cartilage damage continues to influence residual pain following HTO [2]. Comprehensive improvement in clinical symptoms and knee functionality post-HTO generally necessitates a minimum timeframe of six months [3,4]. Given this considerable recovery period, various cartilage regeneration techniques have been performed concurrently with HTO to accelerate the restoration of normal knee function and symptom relief in the medial compartment and extend the survival period after HTO [5,6].

Several cartilage regeneration procedures in HTO have been utilized including microdrilling, autologous chondrocyte implantation (ACI), mesenchymal stem cell (MSC) therapy, bone marrow aspirate concentrate (BMAC), and allogeneic decellularized particulated costal hyaline cartilage allograft (PCHCA) [7,8]. Most of these techniques are host cell-based therapies that are susceptible to the patient’s aging process and cellular senescence, making them more favorable for relatively young patients with localized cartilage defects undergoing HTO [9,10,11]. In contrast, PCHCA combined with microdrilling has been shown to overcome the senescence issues associated with host cell-based therapies while facilitating integration with host mesenchymal stem cells and promoting chondrogenic differentiation [12].

Previous studies have demonstrated that the combination of PCHCA and microdrilling produces superior regenerated cartilage quality compared to microdrilling alone in HTO, as evidenced by improved histological scores and MRI-based cartilage assessment [13,14]. However, these structural improvements have not translated into significant differences in patient-reported clinical outcome scores at mid-term follow-up, suggesting that while both treatments may achieve comparable long-term outcomes, the temporal pattern of recovery during the early postoperative period remains unclear. This distinction is clinically important because patients undergoing HTO typically experience significant pain and functional limitations during the initial 6-month rehabilitation period. Any intervention that accelerates improvement during this challenging phase could substantially enhance patient satisfaction, allow earlier return to activities, and reduce the psychological burden of prolonged recovery. Despite this clinical relevance, previous studies have focused primarily on mid-to-long-term endpoints [15].

Therefore, this prospective randomized controlled trial was designed to address two primary research questions in patients undergoing HTO for medial compartment OA. First, does the addition of PCHCA implantation to microdrilling improve early clinical outcomes during the first 6 months postoperatively compared to microdrilling alone? Second, does patient age influence the treatment effect, specifically comparing outcomes between patients younger than 60 years and those aged 60 years or older?

We hypothesized that combined PCHCA implantation and microdrilling would demonstrate superior clinical score improvement and faster functional recovery compared to microdrilling alone during the critical first six months following HTO. Furthermore, we hypothesized that this beneficial effect would be more pronounced in patients aged 60 years and older, a population with limited cartilage restoration options and age-related decline in intrinsic chondrogenic potential.

## 2. Materials and Methods

### 2.1. Study Design and Patient Selection

This prospective, randomized, participant- and rater-blinded, controlled trial was conducted at two university-affiliated tertiary hospitals in South Korea between March 2023 and August 2025. The study was approved by the Institutional Review Boards at both participating institutions.

Eligible participants were required to meet all of the following criteria: (1) age between 51 and 65 years; (2) provision of written informed consent after receiving detailed explanation of the study; (3) presence of focal full-thickness cartilage defects on medial femoral condyle (MFC) classified as International Cartilage Regeneration and Joint Preservation Society (ICRS) [16] grade IIIB or greater with size of ≥200 mm^2^ and ≤10 cm^2^; (4) isolated symptomatic medial-compartmental OA (Kellgren-Lawrence grade [17] II to III); and (5) patients requiring high tibial osteotomy for varus deformity with HKA angle ≤ −5°, where varus alignment was indicated by a negative value. Patients were excluded if they met any of the following criteria: (1) systemic autoimmune disease or rheumatoid arthritis; (2) trauma or ligament surgery in the same knee within 1 year prior to screening; (3) cartilage injury treatment surgery (microfracture or autologous chondrocyte implantation) in the same knee within 1 year prior to enrollment; (4) intra-articular hyaluronic acid or steroid injection in the same knee within 2 months prior to screening; (5) BMI ≥ 30 kg/m^2^; (6) local infection in the surgical knee; or (7) a minimum follow-up of 6 months after surgery.

Randomization was performed using a centralized computer-generated sequence with permuted blocks of four, administered by an independent research coordinator. Patients and outcome assessors were blinded to group allocation. Surgeons could not be blinded due to the procedural differences between interventions. To preserve blinding, patients were instructed not to discuss surgical details with evaluators.

### 2.2. Surgical Technique

All procedures were performed by three experienced orthopedic surgeons using a uniform surgical technique. Prior to the index procedure, the target correction angle was calculated to position the mechanical axis at approximately 62.5% of the tibial plateau width from the medial border in standing full-length anteroposterior radiographs [18]. Digital measurements were conducted using INFINITT PACS M6 software (INFINITT Healthcare Co., Seoul, Republic of Korea). Initial arthroscopic evaluation was conducted to identify and grade cartilage lesions using the International Cartilage Repair Society (ICRS) Cartilage Repair Assessment grading system. Concomitant arthroscopic interventions, such as intra-articular exploration, loose body extraction, and partial meniscectomy for degenerative meniscal tears, were completed during the HTO procedure.

The surgical approach involved a vertical incision placed superomedially on the proximal tibia, superior to the pes anserinus insertion. A biplanar osteotomy technique was executed following established protocols [19,20] using a medially based locking plate system (TomoFix; Synthes, Solothurn, Switzerland). The osteotomy opening size was based on preoperative planning. Intraoperative verification of alignment correction was performed using fluoroscopic guidance with a long-alignment rod extending from the femoral head center to the ankle center, confirming passage through the predetermined tibial plateau target point. Following plate fixation with locking screws, cancellous allograft bone was applied when the osteotomy gap exceeded 10 mm [21].

Following completion of the osteotomy, access to the medial femoral condyle (MFC) for cartilage repair was achieved through either a mini-arthrotomy incision or arthroscopic portals. The choice between the two approaches was based on the surgeon’s preference. Degenerated cartilage tissue, including fibrillated fragments and the calcified cartilage layer, was removed using ring curettes until viable cartilage margins and subchondral bone were visible. Microdrilling was performed by creating multiple perforations at the cartilage defect site on the MFC. Initial holes (4 mm diameter, 4 mm depth) were drilled into the subchondral plate with 2–3 mm spacing using a 4 mm reamer. Additional smaller perforations were then created between the primary holes using a 2 mm reamer. In the treatment group, cartilage defects were augmented with particulated costal allograft cartilage (MegaCarti, L&C BIO Co., Seongnam, Republic of Korea) in addition to microdrilling. This allograft consists of particulated juvenile costal hyaline cartilage preserved through proprietary processing to maintain viable chondrocytes and extracellular matrix structure. The particulated cartilage pieces (approximately 1–2 mm in diameter) were prepared by thawing the graft at room temperature and gently mixing to achieve uniform consistency.

The prepared PCHCA was carefully press-fitted into the microdrilled holes using a curved spatula and impactor, starting from the deepest portion and progressively filling upward to ensure complete void filling without gaps. Approximately 5–6 cc of PCHCA was utilized per patient, with the exact volume adjusted based on the defect size to achieve filling from the subchondral base to the articular surface level without overfilling. The graft surface was then compressed gently to ensure firm contact with surrounding native cartilage and to create a smooth articular contour.

**Figure 1:**
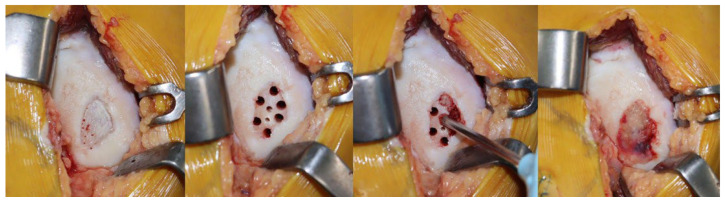
Intraoperative photographs demonstrating the surgical technique. A cartilage defect on the medial femoral condyle after debridement. Multiple microdrilling holes created in the subchondral bone using reamers of various sizes. Particulated costal hyaline cartilage allograft being applied to fill the defect site. Complete coverage of the defect with the cartilage allograft.

Fibrin sealant (Greenplast kit; Green Cross, Seoul, Republic of Korea) was applied circumferentially to seal the interface between the graft and adjacent native cartilage, providing initial mechanical stability and preventing graft displacement. Following a 5 min setting period for the fibrin glue, the knee was passively ranged through full flexion and extension to verify graft stability and ensure no displacement occurred during joint motion before tourniquet release (Figure 1).

### 2.3. Postoperative Management

A standardized rehabilitation regimen was applied uniformly across both treatment and control groups. Immediate postoperative mobilization was initiated, including knee range of motion exercises, manual patellar mobilization, and quadriceps setting exercises beginning on the first postoperative day. Ankle pump exercises were incorporated to facilitate calf muscle activation. Following surgical drain removal on postoperative day 2, patients progressed to both active-assisted and passive range of motion exercises. Machine-assisted range of motion therapy was continued for 4 to 6 weeks postoperatively to maintain joint mobility.

Weight-bearing progression followed a standardized protocol: patients remained non-weight-bearing with bilateral crutch support during the initial postoperative period. Partial weight-bearing ambulation with assistive devices was permitted beginning at 4 to 6 weeks, with advancement to full unprotected weight-bearing allowed between 8 and 12 weeks postoperatively, depending on individual healing progress.

### 2.4. Clinical Assessments

Clinical outcomes were evaluated by independent blinded assessors at postoperative weeks 12 and 24. Assessment tools comprised validated patient-reported outcome measures, specifically the Visual Analog Scale (VAS) for pain intensity, the Knee Injury and Osteoarthritis Outcome Score (KOOS) [22], and the Western Ontario and McMaster Universities Osteoarthritis Index (WOMAC) [23] total score. The primary efficacy endpoint was the KOOS pain subscale score at 24 weeks post-surgery, with scores ranging from 0 to 100. Minimal clinically important difference (MCID) was defined as improvement > 15 points for KOOS-Pain [22] and >10 points for KOOS-ADL [24], based on previously established thresholds for HTO procedures.

### 2.5. Sample Size Calculation

Sample size was calculated based on the primary endpoint of KOOS pain score at 24 weeks. Using a previous study [25] showing mean scores of 72.92 ± 15.54 in the test group versus 65.48 ± 12.48 in the control group, with a pooled standard deviation of 14.09 and mean difference of 7.44 points, the required sample size was 29 patients per group (58 total) using a two-sided significance level of 0.05 and power of 80%. Accounting for a 10% dropout rate, the total required sample size was 64 patients (32 per group).

### 2.6. Statistical Analysis

Continuous variables are presented as mean ± standard deviation (SD), and categorical variables as frequency and percentage. For KOOS, WOMAC, and VAS scores at all time points, descriptive statistics were presented. Between-group comparisons were performed using independent t-tests for continuous variables or Wilcoxon rank-sum test depending on normality (assessed by Shapiro–Wilk test) and chi-square tests (or Fisher’s exact test when appropriate (if >20% of cells had expected frequencies < 5)) for categorical variables. Within-group changes from baseline were tested using paired *t*-test or Wilcoxon signed-rank test.

Multiple logistic regression analysis was conducted to identify factors associated with achieving MCID for KOOS-Pain and KOOS-ADL. Variables included in the model were body mass index (BMI), preoperative hip–knee–ankle (HKA) angle, symptom duration, sex, age (≤60 vs. >60 years), ICRS grade, MFC defect size (≤5 cm^2^ vs. >5 cm^2^), and surgical approach. Results are expressed as odds ratios (OR) with 95% confidence intervals (CI). Statistical significance was set at *p* < 0.05. All analyses were performed using SPSS software (version 26.0; IBM Inc., Armonk, NY, USA, 2019)

## 3. Results

### 3.1. Patient Demographics and Baseline Characteristics

**Figure 2:**
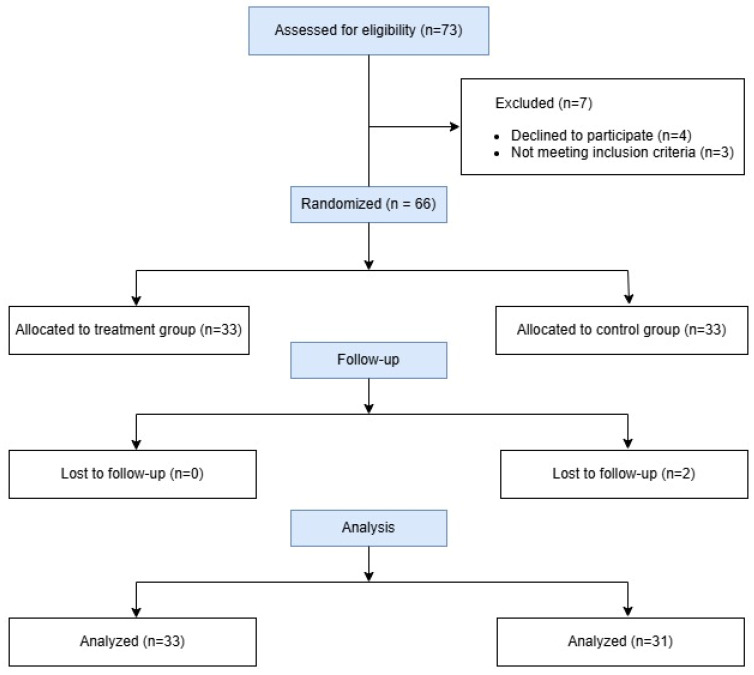
CONSORT Flow Diagram, a flow diagram of participant recruitment, randomization, and follow-up. Seventy-three patients were assessed for eligibility, 66 were randomized, and 64 completed the 6-month follow-up (treatment group n = 33, control group n = 31).

A total of 73 patients were assessed for eligibility from March 2023 to March 2025. Seven patients were excluded due to not meeting inclusion criteria or declining to participate. Sixty-six patients were randomized in a 1:1 ratio to either the treatment group (particulated costal hyaline cartilage allograft with microdrilling, n = 33) or the control group (microdrilling alone, n = 33). Two patients were lost to follow-up, resulting in 64 patients (treatment group (Group T) n = 33, control group (Group C) n = 31) completing the 6-month follow-up and being included in the final analysis (Figure 2).

The demographic and baseline clinical characteristics are summarized in Table 1. No significant differences were observed between the two groups in terms of age (53.4 ± 11.9 years vs. 49.1 ± 11.0 years, *p* = 0.078), sex distribution (*p* = 0.458), body mass index (25.9 ± 4.0 kg/m^2^ vs. 26.8 ± 2.9 kg/m^2^, *p* = 0.229), or symptom duration (11.8 ± 2.1 months vs. 10.9 ± 3.4 months, *p* = 0.283). No significant differences were observed between the two groups in all other variables. At baseline, no significant differences were detected between the treatment and control groups across all outcome measures (all *p* > 0.05), confirming adequate group comparability.

### 3.2. Clinical Outcomes

**Figure 3:**
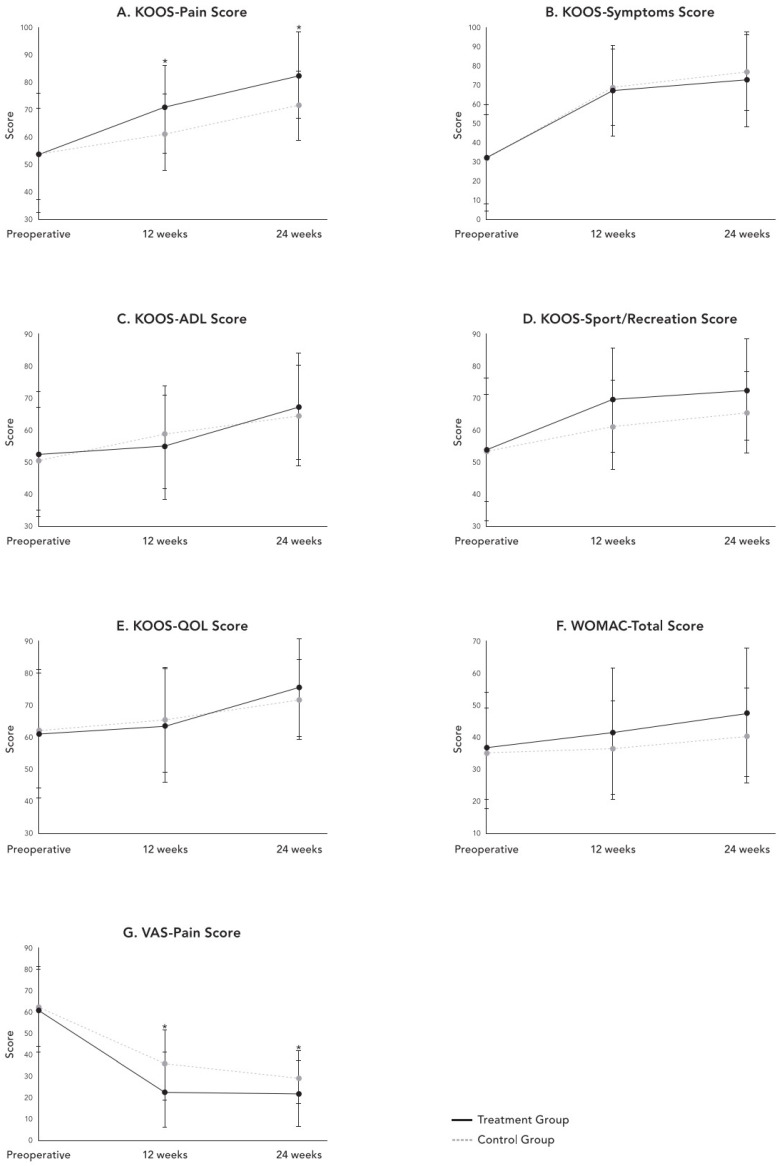
Changes in patient-reported functional outcomes from the preoperative baseline to 12 and 24 weeks postoperatively for the treatment and control groups: (**A**) KOOS-Pain, (**B**) KOOS-Symptoms, (**C**) KOOS-ADL, (**D**) KOOS-Sport/Recreation, (**E**) KOOS-QOL, (**F**) WOMAC-Total, and (**G**) VAS-Pain. Statistically significant differences between groups are indicated by asterisks (*) (*p* < 0.05). ADL, Activities of Daily Living; KOOS, Knee Injury and Osteoarthritis Outcome Score; QOL, Quality of Life; VAS, Visual Analog Scale; and WOMAC, Western Ontario and McMaster Universities Osteoarthritis Index.

At 12 weeks, KOOS-Pain scores were significantly higher (70.6 ± 16.1 vs. 61.6 ± 13.9, *p* = 0.014) and VAS pain scores were significantly lower (23.7 ± 17.9 vs. 35.5 ± 16.4, *p* = 0.010) in the treatment group compared to controls. This advantage persisted and strengthened at 24 weeks, with KOOS-Pain scores of 82.9 ± 15.5 versus 71.5 ± 12.5 (*p* = 0.011) and VAS scores of 21.9 ± 15.7 versus 29.4 ± 12.6 (*p* = 0.004) in the treatment and control groups, respectively. WOMAC total scores approached statistical significance at 24 weeks (47.6 ± 20.3 vs. 40.2 ± 14.9, *p* = 0.055), favoring the treatment group. All other KOOS subscales, including Symptoms, ADL, Sport/Recreation, and QOL, showed no significant intergroup differences at either follow-up time point (all *p* > 0.05), although both groups demonstrated progressive improvement from baseline values (Table 2) (Figure 3).

**Figure 4:**
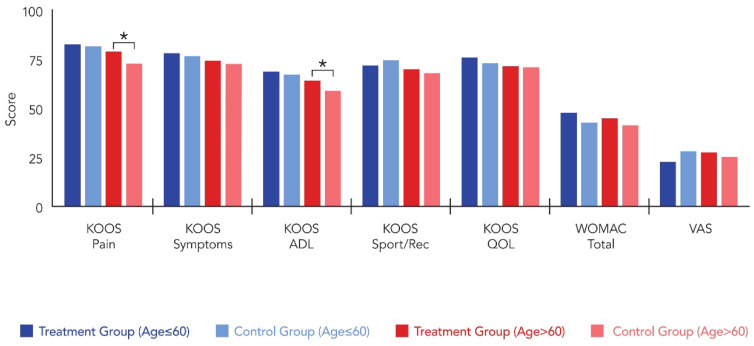
Comparison of postoperative clinical scores between patients aged ≤ 60 and >60 years: 6-months postoperative results. * indicates statistically significant difference (*p* < 0.05).

**Figure 5:**
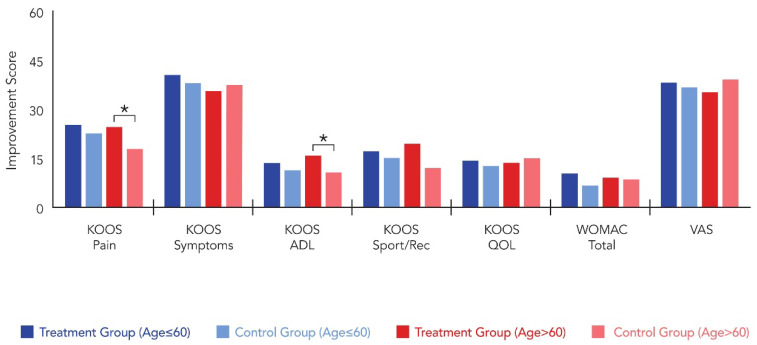
Comparison of postoperative clinical scores between patients aged ≤ 60 and >60 years: improvement from baseline. * indicates statistically significant difference (*p* < 0.05).

In patients aged ≤ 60 years (n = 44), no significant differences were detected between treatment and control groups across all outcome measures. Both groups achieved comparable KOOS-Pain scores (82.8 ± 8.7 vs. 81.7 ± 9.2, *p* = 0.574) and similar improvement from baseline (25.4 ± 11.2 vs. 22.7 ± 10.8, *p* = 0.896). All other functional outcomes, including KOOS subscales, WOMAC total score, and VAS pain scores, remained statistically equivalent between groups (all *p* > 0.05). Conversely, in patients aged > 60 years (n = 20), the treatment group demonstrated significantly superior outcomes in key functional domains. KOOS-Pain scores were significantly higher in Group T at 6 months (79.1 ± 13.3 vs. 72.6 ± 16.1, *p* = 0.025), with significantly greater improvement from baseline (24.7 ± 11.8 vs. 17.9 ± 10.2, *p* = 0.012). KOOS-ADL outcomes similarly favored the treatment group, with higher absolute scores (64.2 ± 18.2 vs. 59.2 ± 16.4, *p* = 0.021) and greater improvement (15.9 vs. 10.8, *p* = 0.014). The remaining KOOS subscales, WOMAC total score, and VAS pain score showed no significant intergroup differences in older patients (all *p* > 0.05) (Table 3) (Figure 4 and Figure 5).

Multiple logistic regression analysis identified BMI and age as the primary predictors of achieving MCID (Table 4). Elevated BMI (≥26 kg/m^2^) was significantly associated with reduced odds of achieving MCID for both KOOS-Pain (OR 0.88, 95% CI 0.81–1.05, *p* = 0.028) and KOOS-ADL (OR 0.80, 95% CI 0.64–1.09, *p* = 0.003), with a stronger effect on functional outcomes. Conversely, age > 60 years was an independent predictor of achieving MCID for pain improvement (OR 1.67, 95% CI 1.12–2.48, *p* = 0.021) and showed a trend for ADL improvement (OR 1.45, *p* = 0.069). Female sex demonstrated trends toward higher odds of achieving MCID for both pain (OR 2.34, *p* = 0.121) and ADL (OR 2.12, *p* = 0.069), though neither reached statistical significance. Arthroscopic approach showed a trend favoring ADL improvement compared to mini-open arthrotomy (OR 3.24, *p* = 0.070). ICRS grade and defect size were not significant predictors of clinical improvement in either domain.

## 4. Discussion

The main finding of this study is that combined particulated costal hyaline cartilage allograft (PCHCA) implantation with microdrilling as a MFC cartilage repair procedure in patients undergoing medial open wedge HTO provided significantly superior pain relief compared to microdrilling alone during the early postoperative period (first 6 months). The treatment group demonstrated significantly better KOOS-Pain scores at both 12 weeks and 24 weeks with corresponding reductions in VAS pain scores. These differences exceed established minimal clinically important difference thresholds, confirming our hypothesis that PCHCA implantation would favorably influence early clinical outcomes within the first six months following HTO. These findings are consistent with the study by Chung et al. [25], which reported that patients treated with costal allograft cartilage showed significantly better patient-reported functional outcomes at 24 weeks in the relatively early postoperative period.

While pain outcomes differed significantly between groups, other functional measures including symptoms, sport/recreation, and quality of life showed comparable improvement, indicating that PCHCA’s early benefit is primarily pain-specific rather than broadly functional. This differential effect may reflect distinct biological timescales: PCHCA provides early pain reduction through mechanical cushioning at the defect site and coverage of pain-sensitive exposed subchondral bone, effects that occur relatively quickly following implantation. In contrast, functional improvements in activities of daily living and sports depend on muscle strength recovery, neuromuscular control, and complete osteotomy healing, requiring extended rehabilitation periods [26,27]. Additionally, the substantial biomechanical correction from HTO itself provides significant functional benefit to both groups, potentially masking incremental improvements from PCHCA during the early period. Longer-term follow-up will be necessary to determine whether early pain reduction translates into sustained functional benefits as cartilage maturation progresses.

Another major finding of this study is the age-dependent treatment effect. While patients aged 60 years or less achieved comparable outcomes regardless of whether PCHCA was added to microdrilling, patients aged over 60 years demonstrated substantially superior results with combined treatment. The older treatment group showed significantly greater improvements in KOOS-Pain and KOOS-ADL compared to microdrilling alone. This differential response validates our hypothesis and suggests that PCHCA implantation effectively compensates for the diminished intrinsic healing capacity characteristic of older patients.

The enhanced outcomes observed with PCHCA implantation in older patients likely reflect several biological mechanisms. PCHCA serves as a scaffold for cartilage regeneration, providing structural support and a favorable microenvironment for chondrocyte proliferation and extracellular matrix synthesis [28,29]. In older patients with reduced cellular proliferation, decreased growth factor expression, and impaired regenerative potential [26], this scaffolding effect may be particularly beneficial. Additionally, PCHCA’s osteoconductive properties may enhance subchondral bone healing following microdrilling, creating a more stable foundation for overlying cartilage repair [30]. The combination of improved mechanical environment from HTO, enhanced marrow stimulation from microdrilling, and biological scaffolding from PCHCA appears to create synergistic effects that are most clinically apparent in the older population where intrinsic healing capacity is limited. For younger patients, various host-cell-dependent cartilage repair strategies including microdrilling, autologous chondrocyte implantation (ACI), mesenchymal stem cell (MSC) therapy, and bone marrow aspirate concentrate (BMAC) may be viable options given their sufficient intrinsic cellular regenerative capacity [31]. However, for older patients, PCHCA implantation represents a promising alternative that addresses the age-related decline in host cell function, providing an acellular scaffold that is less dependent on endogenous cellular activity for cartilage regeneration [32]. This age-stratified approach optimizes resource utilization while maximizing patient-specific outcomes.

The identification of BMI as a negative predictor of MCID achievement has important clinical implications. Higher BMI increases mechanical loading on the regenerating cartilage and osteotomy site, potentially compromising graft integration and healing. Additionally, obesity is associated with systemic low-grade inflammation and altered metabolic environment that may impair cartilage regeneration. The dose–response relationship (decreasing odds with each BMI unit increase) suggests that weight optimization prior to surgery may improve outcomes following PCHCA implantation. Interestingly, an age older than 60 years positively predicted pain improvement in the treatment group, supporting our hypothesis that PCHCA provides particular benefit in older patients with limited autologous cartilage restoration options. This age-related advantage may reflect the allogeneic nature of PCHCA, which bypasses the age-related decline in autologous chondrogenic potential. These findings suggest that patient selection based on BMI and age could optimize treatment outcomes.

The stronger effect on functional outcomes (20% reduction in odds) compared to pain outcomes (12% reduction) indicates that elevated BMI particularly impedes the recovery of activities of daily living. This BMI-outcome relationship likely reflects multiple interconnected mechanisms. Biomechanically, higher BMI increases joint loading forces, potentially overwhelming the benefits of HTO correction and compromising PCHCA scaffold integration [33]. The increased mechanical stress may impair healing at both the cartilage repair site and osteotomy site [34]. From a biological perspective, obesity is associated with chronic low-grade systemic inflammation characterized by elevated pro-inflammatory cytokines (TNF-α, IL-6) that create a hostile microenvironment for cartilage regeneration [35]. This inflammatory state may impair chondrocyte proliferation within the PCHCA scaffold and reduce extracellular matrix synthesis. Additionally, patients with higher BMI may experience reduced compliance with postoperative rehabilitation protocols and weight-bearing restrictions, indirectly affecting functional outcomes [36].

Age over 60 years was a significant independent predictor of achieving MCID for pain improvement (OR 1.67, 95% CI 1.12–2.48, *p* = 0.021), though it did not reach significance for ADL improvement (OR 1.45, *p* = 0.069). Being over the age of 60 does not inherently confer better prognosis, but rather reflects the differential treatment response demonstrated in Table 3. In patients ≤ 60 years, both treatment and control groups achieved nearly identical outcomes, with KOOS-Pain scores of 82.8 ± 8.7 versus 81.7 ± 9.2 (*p* = 0.574) and comparable improvements from baseline (25.4 ± 11.2 vs. 22.7 ± 10.8 points, *p* = 0.896). In contrast, patients > 60 years demonstrated markedly divergent outcomes between groups. Control group patients (microdrilling alone) achieved KOOS-Pain scores of only 72.6 ± 16.1 with 17.9 ± 10.2 points of improvement, significantly inferior to their younger counterparts. However, older patients receiving PCHCA achieved KOOS-Pain scores of 79.1 ± 13.3 with 24.7 ± 11.8 points of improvement (*p* = 0.025 and *p* = 0.012, respectively, versus control), restoring outcomes to levels comparable with younger patients. Similarly, KOOS-ADL outcomes in older controls were substantially compromised (59.2 ± 16.4, improvement 10.8 points), while PCHCA-treated older patients achieved significantly better results (64.2 ± 18.2, improvement 15.9 points; *p* = 0.021 and *p* = 0.014 respectively).

Therefore, this regression result indicates that older patients derive disproportionate benefit from PCHCA augmentation—not because aging improves prognosis, but because PCHCA effectively compensates for age-related decline in healing capacity that profoundly limits outcomes with microdrilling alone. This rescues older patients from the poor outcomes observed in controls, making them significantly more likely to achieve MCID when appropriately treated with PCHCA.

### Limitations

The study limitations include its modest sample size, particularly in the older subgroup (n = 20), its relatively short follow-up duration, limiting the assessment of cartilage regeneration quality and long-term durability, and the absence of imaging outcomes to correlate clinical improvement with structural cartilage repair. The lack of imaging follow-up and second-look arthroscopy precluded direct assessment of cartilage regeneration quality and integration, representing important structural outcome measures that could not be evaluated in this study. Future research should include longer-term follow-up with imaging evaluation to assess cartilage fill and integration, histological or second-look arthroscopic evaluation where feasible, and cost-effectiveness analysis comparing age-stratified treatment algorithms. Additional limitations include the inability to blind surgeons to group allocation due to the nature of the surgical procedures, and potential variability in surgeon experience and case volume between the two participating centers, which may have influenced treatment outcomes.

## 5. Conclusions

In conclusion, the present findings suggest that combined PCHCA augmentation with microdrilling may be associated with improved early pain relief compared to micro drilling alone following MOWHTO, particularly in patients aged over 60 years. Age-stratified treatment selection and consideration of BMI may be important factors when applying PCHCA augmentation in the setting of MOWHTO.

## Figures and Tables

**Table 1:** Patient demographics and baseline characteristics.

Variables	Group T (n = 33)	Group C (n = 31)	*p*-Value
Age (years) ^†^	53.4 ± 11.9	49.1 ± 11	0.078
≤60	22 (66.7%)	22 (70.9%)	
>60	11 (33.3%)	9 (29.1%)	
Sex ^‡^			0.458
Male	7 (21.2%)	10 (32.3%)	
Female	26 (78.3%)	21 (67.7%)	
Body mass index (kg/m^2^) ^†^	25.9 ± 4	26.8 ± 2.9	0.229
Symptom duration (months) ^†^	11.8 ± 2.1	10.9 ± 3.4	0.283
Cartilage defect size on MFC, cm^2 †^	5.1 ± 2.7	5.9 ± 2.4	0.639
ICRS grade on MFC defect site ^‡^			0.415
Grade III	13 (39.4%)	14 (45.2%)	
Grade IV	20 (60.6%)	17 (54.8%)	
Bone graft, n (%) ^‡^	10 (30.3%)	12 (38.7%)	0.715
Preoperative HKA angle ^†^	−7.4 ± 3.9	−6.8 ± 2.7	0.430
Correction Angle, degree (°) ^†^	10.2 ± 2.8	9.4 ± 2.1	0.274
Approach ^‡^			0.617
Mini-Open arthrotomy	16 (48.5%)	4 (25.8%)	
Arthroscopy	17 (51.5%)	27 (74.2%)	

NOTE. The level of statistical significance was set at *p* < 0.05. Data are presented as number or mean (standard deviation). Varus alignment was indicated as a negative value. MFC, Medial Femoral Condyle; ICRS, International Cartilage Regeneration and Joint Preservation Society; and HKA, Hip–Knee–Ankle. ^†^ The values are given as mean ± standard deviation. ^‡^ The values are given as n (%). Group T: treatment group. Group C: control group.

**Table 2:** Comparison of functional outcomes between the treatment and control groups at each follow-up.

	Preoperatively			At 12 Weeks			At 24 Weeks		
Variable	Treatment	Control	*p*	Treatment	Control	*p*	Treatment	Control	*p*
KOOS—Pain ^†^	54.4 ± 21.5	54.2 ± 16.5	0.964	70.6 ± 16.1	61.6 ± 13.9	**0.014**	82.9 ± 15.5	71.5 ± 12.5	**0.011**
KOOS—Symptoms ^†^	32.3 ± 27.6	31.4 ± 23.0	0.831	66.9 ± 23.5	68.6 ± 19.5	0.293	73.4 ± 24.8	76.6 ± 20.1	0.156
KOOS—ADL ^†^	53.1 ± 19.5	51.0 ± 15.9	0.577	55.1 ± 16.5	58.3 ± 15.9	0.392	67.7 ± 16.3	64.7 ± 16.0	0.386
KOOS—Sport/Rec ^†^	54.4 ± 21.5	54.2 ± 16.5	0.964	69.6 ± 16.1	61.6 ± 13.9	0.314	72.9 ± 15.5	65.5 ± 12.5	0.571
KOOS—QOL ^†^	61.4 ± 19.9	62.4 ± 17.8	0.803	63.7 ± 17.9	65.5 ± 16.4	0.712	75.9 ± 15.7	71.9 ± 12.6	0.127
WOMAC—Total ^†^	36.1 ± 18.3	35.0 ± 14.2	0.913	42.2 ± 20.0	36.0 ± 15.6	0.256	47.6 ± 20.3	40.2 ± 14.9	0.055
VAS ^†^	61.4 ± 19.9	62.4 ± 17.8	0.803	23.7 ± 17.9	35.5 ± 16.4	**0.010**	21.9 ± 15.7	29.4 ± 12.6	**0.004**

^†^ The values are presented as mean ± standard deviation. Bold *p*-values indicate statistical significance (*p* < 0.05). KOOS, Knee Injury and Osteoarthritis Outcome Score; ADL, Activities of Daily Living; QOL, Quality of Life; WOMAC, Western Ontario and McMaster Universities Osteoarthritis Index; and VAS, Visual Analog Scale.

**Table 3:** Age-Stratified Comparison of Functional Outcomes at 6 Months Postoperatively.

Variables	Under 60			Over 60		
	Group T (n = 22)	Group C (n = 22)	*p*-Value	Group T (n = 11)	Group C (n = 9)	*p*-Value
KOOS—Pain ^†^						
Postop 6M	82.8 ± 8.7	81.7 ± 9.2	0.574	79.1 ± 13.3	72.6 ± 16.1	**0.025**
Improvement	25.4 ± 11.2	22.7 ± 10.8	0.896	24.7 ± 11.8	17.9 ± 10.2	**0.012**
KOOS—Symptoms ^†^						
Postop 6M	78.2 ± 11.2	76.8 ± 10.4	0.667	74.3 ± 13.4	72.9 ± 14.9	0.839
Improvement	40.4 ± 12.7	38.1 ± 21.4	0.789	35.6 ± 19.2	37.4 ± 20.1	0.576
KOOS—ADL ^†^						
Postop 6M	68.9 ± 14.4	67.2 ± 15.1	0.451	64.2 ± 18.2	59.2 ± 16.4	**0.021**
Improvement	13.7 ± 9.1	11.4 ± 8.8	0.367	15.9	10.8	**0.014**
KOOS—Sport/Rec ^†^						
Postop 6M	72.1 ± 13.7	74.7 ± 11.4	0.134	69.8 ± 16.1	68.2 ± 15.4	0.761
Improvement	17.2 ± 6.4	15.1 ± 9.7	0.561	19.4 ± 5.7	12.1 ± 6.3	0.403
KOOS—QOL ^†^						
Postop 6M	75.8 ± 14.6	73.2 ± 13.6	0.356	71.4 ± 13.8	70.9 ± 14.7	0.181
Improvement	14.3 ± 7.1	12.7 ± 6.9	0.256	13.6 ± 4.8	15.1 ± 5.5	0.518
WOMAC– Total ^†^						
Postop 6M	47.9 ± 6.8	42.8 ± 8.3	0.077	44.9 ± 14.1	41.6 ± 17.2	0.204
Improvement	10.4 ± 7.3	6.7 ± 8.9	0.156	9.2 ± 5.4	8.6 ± 5.7	0.079
VAS						
Postop 6M	22.7 ± 8.4	28.4 ± 7.9	0.192	27.7 ± 16.1	25.4 ± 17.1	0.061
Improvement	38.2 ± 20.1	36.7 ± 16.4	0.803	35.4 ± 14.9	39.1 ± 13.0	0.504

^†^ The values are presented as mean ± standard deviation. Bold *p*-values indicate statistical significance (*p* < 0.05).

**Table 4:** Factors Associated with Achieving Minimal Clinically Important Difference in KOOS Subscales (Multivariable Logistic Regression).

Variables	KOOS-Pain Improvement > 15		KOOS-ADL Improvement > 10	
	OR (95% CI)	*p*-Value	OR (95% CI)	*p*-Value
**BMI (kg/m^2^)**				
<26	1.00 (Reference)		1.00 (Reference)	
≥26	0.879(0.81–1.05)	**0.028**	0.798 (0.64–1.09)	**0.003**
**Sex**				
Male	1.00 (Reference)		1.00 (Reference)	
Female	2.34 (1.45–3.78)	0.121	2.12 (1.28–3.51)	0.069
**Age**				
≤60	1.00 (Reference)		1.00 (Reference)	
>60	1.67 (1.12–2.48)	**0.021**	1.45 (0.94–2.24)	0.069
**ICRS grade on MFC defect site**				
ICRS GIII	1.00 (Reference)		1.00 (Reference)	
ICRS IV	0.892 (0.72–1.14)	0.213	0.913 (0.89–1.23)	0.069
**Defect size of MFC**				
≤5 cm^2^	1.00 (Reference)		1.00 (Reference)	
>5 cm^2^	1.23 (0.78–1.94)	0.377	0.89 (0.58–1.24)	0.602
**Approach**				
Mini-Open arthrotomy	1.00 (Reference)		1.00 (Reference)	
Arthroscopy	2.86 (0.757–10.847)	0.41	3.24 (0.913–11.248)	0.07

*p*-values indicate statistical significance (*p* < 0.05). OR, Odds Ratio; CI, Confidence Interval; KOOS, Knee Injury and Osteoarthritis Outcome Score; ADL, Activities of Daily Living; ICRS, International Cartilage Repair Society; and MFC, Medial Femoral Condyle.

## Data Availability

The data supporting the findings of this study are not publicly available due to ethical and privacy considerations, but may be available from the corresponding author upon reasonable request.

## References

[1] Schuster P., Geßlein M., Schlumberger M., Mayer P., Mayr R., Oremek D., Frank S., Schulz-Jahrsdörfer M., Richter J. (2018). Ten-Year Results of Medial Open-Wedge High Tibial Osteotomy and Chondral Resurfacing in Severe Medial Osteoarthritis and Varus Malalignment. Am. J. Sports Med..

[2] Danese I., Pankaj P., Scott C.E.H. (2019). The Effect of Malalignment on Proximal Tibial Strain in Fixed-Bearing Unicompartmental Knee Arthroplasty. Bone Jt. Res..

[3] Peeters G., Munck Id Tooth L., Melis R.J.F. (2024). Quantifying Physical Resilience After Knee or Hip Surgery in Older Australian Women Based on Long-Term Physical Functioning Trajectories. Gerontology.

[4] Li Y., Zhang H., Zhang J., Li X., Song G., Feng H. (2015). Clinical outcome of simultaneous high tibial osteotomy and anterior cruciate ligament reconstruction for medial compartment osteoarthritis in young patients with anterior cruciate ligament-deficient knees: A systematic review. Arthroscopy.

[5] Tan S.H.S., Kwan Y.T., Neo W.J., Chong J.Y., Kuek T.Y.J., See J.Z.F., Hui J.H. (2021). Outcomes of High Tibial Osteotomy With Versus Without Mesenchymal Stem Cell Augmentation: A Systematic Review and Meta-Analysis. Orthop. J. Sports Med..

[6] Mabrouk A., An J.S., Kley K., Tapasvi K., Tapasvi S., Ollivier M. (2024). Combined Knee Osteotomy and Cartilage Procedure for Varus Knees: Friend or Foe? A Narrative Review of the Literature. Efort Open Rev..

[7] Han J.H., Jung M., Chung K., Jung S.H., Choi C.H., Kim S.H. (2024). Effects of concurrent cartilage procedures on cartilage regeneration in high tibial osteotomy: A systematic review. Knee Surg. Relat. Res..

[8] Lee D.H., Kim S.J., Kim S.A., Ju G.I. (2022). Past, present, and future of cartilage restoration: From localized defect to arthritis. Knee Surg. Relat. Res..

[9] Li D., Gao F.-H., Wu C.-F., Liang Z.-J., Xiong W. (2021). miR-34a/SIRT1 Axis Plays a Critical Role in Regulating Chondrocyte Senescence in Type 2 Diabetes Mellitus. Explor. Res. Hypothesis Med..

[10] Wang D., Dai J., Shen X., Zhu J., Pang X., Lin Z., Tang B. (2024). Regulating Chondrocyte Senescence Through Substrate Stiffness Modulation. J. Appl. Polym. Sci..

[11] Park Y.-B., Lee H.-J., Nam H.-C., Park J.-G. (2023). Allogeneic umbilical cord-blood-derived mesenchymal stem cells and hyaluronate composite combined with high tibial osteotomy for medial knee osteoarthritis with full-thickness cartilage defects. Medicina.

[12] Lohan P., Treacy O., Lynch K., Barry F., Murphy M., Griffin M.D., Ritter T., Ryan A. (2016). Culture Expanded Primary Chondrocytes Have Potent Immunomodulatory Properties and Do Not Induce an Allogeneic Immune Response. Osteoarthr. Cartil..

[13] Chung K., Jung M., Jang K.M., Park S., Kim J., Kim S.H. (2025). Acellular Particulated Costal Allocartilage Improves Cartilage Regeneration in High Tibial Osteotomy: Data From a Randomized Controlled Trial. Cartilage.

[14] Shon O.-J., On J.W., Kim G.B. (2023). Particulated costal hyaline cartilage allograft with subchondral drilling improves joint space width and second-look macroscopic articular cartilage scores compared with subchondral drilling alone in medial open-wedge high tibial osteotomy. Arthrosc. J. Arthrosc. Relat. Surg..

[15] Bode G., Heyden Jv Pestka J.M., Schmal H., Salzmann G.M., Südkamp N.P., Niemeyer P. (2013). Prospective 5-year Survival Rate Data Following Open-wedge Valgus High Tibial Osteotomy. Knee Surg. Sports Traumatol. Arthrosc..

[16] Anwar W., Aziz Z., Rahman N., Khattak S.K., Ahmad I., Khan A.H. (2023). Arthroscopic Evaluation of Articular Cartilage in Knee Injuries: A Predictor of Early Osteoarthritis in Young Population. Prof. Med. J..

[17] Boldbayar T., Sosor B., Maidar O., Orgoi S., Dagvasumberel M. (2022). Mid-Term Results of High Tibial Osteotomy Regarding From Grades of Knee Osteoarthritis. Cent. Asian J. Med. Sci..

[18] Feucht M.J., Winkler P.W., Mehl J., Bode G., Forkel P., Imhoff A.B., Lutz P.M. (2020). Isolated High Tibial Osteotomy Is Appropriate in Less Than Two-thirds of Varus Knees if Excessive Overcorrection of the Medial Proximal Tibial Angle Should Be Avoided. Knee Surg. Sports Traumatol. Arthrosc..

[19] Zheng Y., Wang Z., Lv H., Li J., Zhuo R., Wang J. (2022). Patellofemoral Joint after Opening Wedge High Tibial Osteotomy: A Comparative Study of Uniplane versus Biplane Osteotomies. Orthop. Surg..

[20] Lobenhoffer P., Agneskirchner J.D. (2003). Improvements in surgical technique of valgus high tibial osteotomy. Knee Surg. Sports Traumatol. Arthrosc..

[21] Brinkman J.M., Lobenhoffer P., Agneskirchner J.D., Staubli A.E., Wymenga A.B., van Heerwaarden R.J. (2008). Osteotomies around the knee: Patient selection, stability of fixation and bone healing in high tibial osteotomies. J. Bone Jt. Surg. Br..

[22] Jacquet C., Pioger C., Khakha R., Steltzlen C., Kley K., Pujol N., Ollivier M. (2021). Evaluation of the “Minimal Clinically Important Difference” (MCID) of the KOOS, KSS and SF-12 scores after open-wedge high tibial osteotomy. Knee Surg. Sports Traumatol. Arthrosc..

[23] Kim S.E., Ro D.H., Lee M.C., Han H.S. (2024). Can individual functional improvements be predicted in osteoarthritic patients after total knee arthroplasty?. Knee Surg. Relat. Res..

[24] Retzky J.S., Shah A.K., Neijna A.G., Rizy M., Gomoll A.H., Strickland S.M. (2024). Defining the Minimal Clinically Important Difference for IKDC and KOOS Scores for Patients Undergoing Tibial Tubercle Osteotomy for Patellofemoral Pain or Instability. J. Exp. Orthop..

[25] Chung K., Jung M., Jang K.M., Park S.H., Nam B.J., Kim H., Kim S.-H. (2023). Particulated Costal Allocartilage With Microfracture Versus Microfracture Alone for Knee Cartilage Defects: A Multicenter, Prospective, Randomized, Participant- and Rater-Blinded Study. Orthop. J. Sports Med..

[26] Choi I.-S., Ingle P.S., Seon J.K., Song E.K., Jin Q.-H., Na S.-M. (2019). Risk Factors of Poor Cartilage Regeneration in Patients Who Underwent High Tibial Osteotomy Combined With Microfracture. Res. Sq..

[27] Zhou G., Jiang H., Yin Z., Liu Y., Zhang Q., Zhang C., Pan B., Zhou J., Zhou X., Sun H. (2018). In Vitro Regeneration of Patient-Specific Ear-Shaped Cartilage and Its First Clinical Application for Auricular Reconstruction. Ebiomedicine.

[28] Yang Z., Li H., Tian Y., Fu L., Gao C., Zhao T., Cao F., Liao Z., Yuan Z., Liu S. (2021). Biofunctionalized Structure and Ingredient Mimicking Scaffolds Achieving Recruitment and Chondrogenesis for Staged Cartilage Regeneration. Front. Cell Dev. Biol..

[29] Nishiwaki H., Fujita M., Yamauchi M., Isogai N., Tabata Y., Kusuhara H. (2017). A Novel Method to Induce Cartilage Regeneration With Cubic Microcartilage. Cells Tissues Organs.

[30] Gwosdz J., Rosinski A., Chakrabarti M., Woodall B.M., Elena N., McGahan P.J., Chen J.L. (2019). Osteochondral Allograft Transplantation of the Medial Femoral Condyle With Orthobiologic Augmentation and Graft-Recipient Microfracture Preparation. Arthrosc. Tech..

[31] Kim K.I., Kim J.H., Lee S.H., Song S.J., Jo M.-G. (2022). Mid- To Long-Term Outcomes After Medial Open-Wedge High Tibial Osteotomy in Patients With Radiological Kissing Lesion. Orthop. J. Sports Med..

[32] Cugat R., Samitier G., Vinagre G., Sava M., Alentorn-Geli E., García-Balletbó M., Cuscó X., Seijas R., Barastegui D., Navarro J. (2021). Particulated Autologous Chondral−Platelet-Rich Plasma Matrix Implantation (PACI) for Treatment of Full-Thickness Cartilage Osteochondral Defects. Arthrosc. Tech..

[33] Pieri E.D., Nüesch C., Pagenstert G., Viehweger E., Egloff C., Mündermann A. (2022). High Tibial Osteotomy Effectively Redistributes Compressive Knee Loads During Walking. J. Orthop. Res.^®^.

[34] Wang S., Bao Y., Guan Y., Zhang C., Liu H., Yang X., Gao L., Guo T., Chen Q. (2018). Strain Distribution of Repaired Articular Cartilage Defects by Tissue Engineering Under Compression Loading. J. Orthop. Surg. Res..

[35] Hp F., Bakri S. (2020). Obesity Contribution in Synthesis and Degradation of Cartilage Marker Through Inflammation Pathway in Osteoarthritis Patient: Analysis of Adiponectin, Leptin, Ykl-40 and Cartilage Oligomeric Matrix Proteinase (Comp) Synovial Fluid. Indian. J. Public Health Res. Dev..

[36] Rucinski K., Cook J.L., Crecelius C.R., Stucky R., Stannard J.P. (2019). Effects of Compliance With Procedure-Specific Postoperative Rehabilitation Protocols on Initial Outcomes After Osteochondral and Meniscal Allograft Transplantation in the Knee. Orthop. J. Sports Med..

